# Autocrine selection of a GLP-1R G-protein biased agonist with potent antidiabetic effects

**DOI:** 10.1038/ncomms9918

**Published:** 2015-12-01

**Authors:** Hongkai Zhang, Emmanuel Sturchler, Jiang Zhu, Ainhoa Nieto, Philip A. Cistrone, Jia Xie, LinLing He, Kyungmoo Yea, Teresa Jones, Rachel Turn, Peter S. Di Stefano, Patrick R. Griffin, Philip E. Dawson, Patricia H. McDonald, Richard A. Lerner

**Affiliations:** 1 Department of Cell and Molecular Biology, The Scripps Research Institute, La Jolla, California 92037, USA; 2 Department of Molecular Therapeutics, The Scripps Research Institute, Jupiter, Florida 33458, USA; 3 Department of Immunology and Microbial Science, The Scripps Research Institute, La Jolla, California 92037, USA; 4 Department of Chemistry, The Scripps Research Institute, La Jolla, California 92037, USA; 5 Shanghai Institute for Advance Immunological Studies, Shanghai Tech University, Shanghai 200031, China; 6 Zebra Biologics Inc., Concord, Massachusetts 01742, USA

## Abstract

Glucagon-like peptide-1 (GLP-1) receptor (GLP-1R) agonists have emerged as treatment options for type 2 diabetes mellitus (T2DM). GLP-1R signals through G-protein-dependent, and G-protein-independent pathways by engaging the scaffold protein β-arrestin; preferential signalling of ligands through one or the other of these branches is known as ‘ligand bias'. Here we report the discovery of the potent and selective GLP-1R G-protein-biased agonist, P5. We identified P5 in a high-throughput autocrine-based screening of large combinatorial peptide libraries, and show that P5 promotes G-protein signalling comparable to GLP-1 and Exendin-4, but exhibited a significantly reduced β-arrestin response. Preclinical studies using different mouse models of T2DM demonstrate that P5 is a weak insulin secretagogue. Nevertheless, chronic treatment of diabetic mice with P5 increased adipogenesis, reduced adipose tissue inflammation as well as hepatic steatosis and was more effective at correcting hyperglycaemia and lowering haemoglobin A_1c_ levels than Exendin-4, suggesting that GLP-1R G-protein-biased agonists may provide a novel therapeutic approach to T2DM.

## Abstract (web summary)

GLP-1 is a gut hormone with glucose-lowering activity. Here the authors report the peptide, P5, a variant of the GLP-1 receptor agonist exendin-4, with 'biased' signalling activity, and show that P5 improves glucose homeostasis in diabetic mice by increasing adipose tissue hyperplasia.

## Graphical abstract

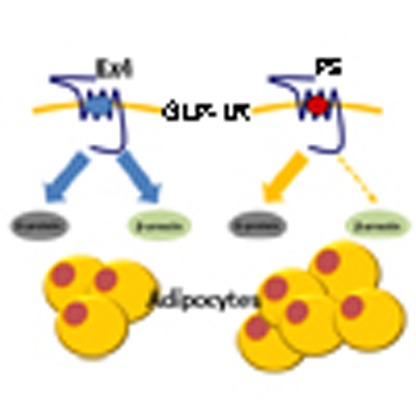

Type 2 diabetes mellitus (T2DM) is a complex metabolic disorder characterized by hyperglycaemia arising from a combination of insufficient insulin secretion together with the development of insulin resistance. The incretin, glucagon-like peptide-1 (GLP-1) is an endogenous peptide hormone secreted from intestinal endocrine cells in response to food intake^1^. GLP-1 lowers postprandial glucose excursion by potentiating glucose-stimulated insulin secretion from pancreatic β-cells and has also recently been shown to promote β-cell survival in rodents^2^. In addition, GLP-1 exerts extra-pancreatic actions such as promoting gastric emptying, weight loss and increasing insulin sensitivity in peripheral tissues^3^. Hence, incretin-based therapies represent a strategy for the treatment of T2DM.

GLP-1 exerts its action through the GLP-1 receptor (GLP-1R)^4^ expressed in the pancreas, other peripheral tissues, and the central nervous system. Activation of GLP-1R triggers Gαs-protein coupling leading to an elevation of cyclic AMP (cAMP), modulates intracellular calcium concentration^5^ and induces β-arrestin recruitment^6^,^7^. Historically, β-arrestins were believed to serve an exclusive role in G-protein coupled receptor (GPCR) desensitization^8^. However, it has since been shown that β-arrestins can also function to activate signalling cascades^9^,^10^. In this regard, in the pancreatic β-cell, elevation of both cAMP and cytosolic Ca^2+^ and β-arrestin signalling downstream of GLP-1R activation are critical events in promoting glucose-dependent insulin secretion.

Recently, the concept of ‘functional selectivity' or ‘ligand bias' has emerged whereby ligand binding promotes engagement of only a particular subset of the full GPCR signalling repertoire to the exclusion of others^11^. A better understanding of GLP-1R pleiotropic signalling and the underlying physiological consequences might provide new avenues for the development of drugs with novel modes of action that have the potential to provide greater therapeutic value while possibly avoiding unwanted side effects^12^,^13^. Therefore we developed an autocrine-based system, to screen large and diverse, combinatorial peptide libraries containing up to 100 million different members with the aim of identifying potent, selective, G-protein-biased GLP-1R agonists. We identified one such ligand, designated P5 and have characterized its *in vitro* pharmacological phenotype, and explored its therapeutic potential.

## Results

### Autocrine-based selection of a novel GLP-1R agonist

To identify potential G-protein-biased ligands for the GLP-1R we expressed a peptide library at the cell surface of a reporter cell line co-expressing the GLP-1R and the green fluorescent protein (GFP) reporter gene under the control of a CRE promoter (HEK293-GLP-1R-GFP) to screen for peptides that induce GLP-1R-mediated cAMP production. This autocrine system coupled to fluorescence-activated cell sorting (FACS) has the capability to screen as many as 20 million events per hour. The Exendin-4 (Ex4) is a 39 amino acid peptide agonist of the GLP-1 receptor. The C-terminal regions of Ex4 interact with the N terminus extracellular domain of the GLP-1R, facilitating the interaction of the Ex4 N terminus with the transmembrane domain of the receptor leading to receptor activation^3^. In contrast, the peptide Exendin 9–39 (Ex 9–39; Ex4 lacking the 8 first amino acids) behaves as a competitive antagonist^14^. On the basis of these Exendin structure–activity insights, three combinatorial peptide libraries consisting of random 7–10 amino acids fused to the N terminus of Ex 9–39 were generated.

**Figure 1:**
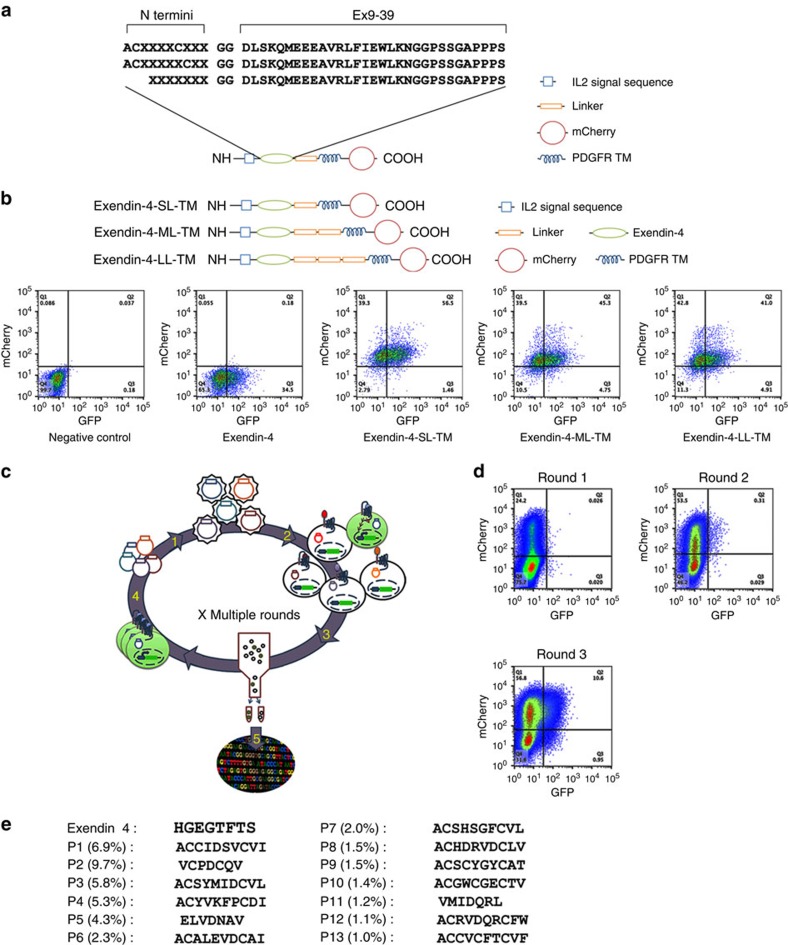
Autocrine-based system for selection of agonists from large combinatorial peptide libraries. (**a**) Schematic representation of the peptide libraries. (**b**) Schematic representation of the membrane-tethered Exendin-4 (top) and FACS analysis of mCherry and GFP expression 2 days after transduction of HEK293-GLP-1R-GFP cells with the membrane-tethered Exendin-4 displaying different linker size (bottom). (**c**) Schematic representation of the autocrine-based selection of combinatorial peptide library. The lentivirus peptide libraries are preparred from lentiviral plasmids (step 1). The CRE-responsive GLP-1R reporter cell line is transduced with lentiviral library (step 2). GFP expressing cells are sorted (step 3) and peptide-encoding genes are amplified from genomic DNA of sorted cells to make the library for the next selection round (step 4). After iterative rounds of selection, enriched peptide sequences are analysed by deep sequencing (step 5). (**d**) Enrichment of GFP positive cells during three rounds of FACS selection. (**e**) N termini sequences of top 13 peptides (frequency>1.0% representation).

As several GPCR natural ligands are cyclic peptides such as calcitonin, oxytocin and somatostatin^15^, two libraries encoding cyclic peptide N termini (either randomized tetrapeptide (CX_4_C), or pentapeptide (CX_5_C)) were designed (Fig. 1a). The third library consisted of seven random amino acids fused to the N terminus of the Ex 9–39 (Fig. 1a). Each library was inserted between a cleavable interleukin 2 signal sequence used to promote cell surface localization and a linker followed by the platelet-derived growth factor receptor (PDGFR) transmembrane domain to anchor the library at the cell surface (Fig. 1a). In addition, the mCherry fluorescent protein was fused to the C terminus of the PDGFR sequence to enable monitoring of transduction efficiency.

To determine the optimal length of the linker between the peptide sequence and the PDGFR transmembrane anchor we first infected HEK293-GLP-1R-GFP cells with lentivirus encoding membrane-tethered Ex4 containing one, two or three repeats of the Gly–Gly–Gly–Gly–Ser linker sequence (Fig. 1b, top panel). Tethered Ex4-mediated GLP-1R activation as determined by FACS analysis was found to be independent of the length of the linker between the PDGFR transmembrane domain and the peptide library (Fig. 1b, bottom panel). Therefore, the three libraries described above were inserted upstream of a single linker sequence followed by the PDGFR anchor for large peptide library screening (Fig. 1a). Following lentiviral infection of the HEK293-GLP-1R-GFP cell line with the peptide libraries (encoding ∼10^6^ distinct peptides), active peptides were selected by multiple rounds of enrichment using our standard protocol^16^ (Fig. 1c). To achieve single copy integration, a multiplicity of infection <1 was used. Following each round of selection, genes encoding the active peptides were recovered from sorted cells by PCR and subcloned into a lentiviral vector to generate an enriched library used for the next round of selection. Following the first round, no significant enrichment could be detected (Fig. 1d). However, following a second round, enrichment was observed (0.31% of the population) and by the third round of selection, 10.6% of the cells were GFP positive (Fig. 1d). Using deep sequencing, 13 peptide sequences with frequency higher than 1% were identified following the third round of selection and considered as hits (Fig. 1e). Notably, we identified putative agonist sequences from the linear heptapeptide (X_7_) and the heptapeptide (CX_5_C) library but not from the hexapeptide library (CX_4_C). Strikingly, all 13 sequences were unrelated to the N terminus of the parent molecule Ex4.

### P5 is a selective and potent G-protein-biased GLP-1R agonist

**Figure 2:**
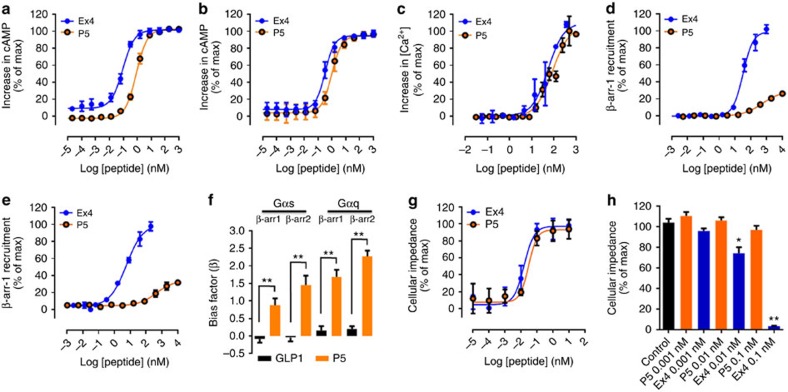
***In vitro*** **pharmacological characterization of P5 (ELVDNAVGGDLSKQMEEEAVRLFIEWLKNGGPSSGAPPPS).** (**a**,**b**) Concentration–response curves for P5- and Ex4-induced increase in cAMP production in CHO cells expressing the human GLP-1R (**a**) or in HEK293 cells expressing the mouse GLP-1R (**b**). (**c**) Concentration–response curves for P5, and Ex4-induced calcium mobilization in CHO cells expressing the human GLP-1R. (**d**,**e**) Concentration–response curves for P5, and Ex4-induced β-arrestin 1 (**d**) and β-arrestin 2 (**e**) recruitment in CHO cells expressing the human GLP-1R. (**f**) Biased factors (*β*) from an equiactive comparison indicate bias for P5. (**g**) Concentration–response curves for P5- and Ex4-induced changes in cellular impedance in CHO cells expressing the human GLP-1R. (**h**) Concentration-dependent effects of P5 and Ex4 toward GLP-1R desensitization (*n*=3). The data are mean±s.e.m. of a typical experiment that was performed independently at least three times. Statistic by two-tailed *t*-test: *P<0.05; **P<0.01.

To assess potential signalling bias, the active peptides were further characterized *in vitro* using distinct assays that monitor receptor proximal signals. Cell-based assays for Gαs-protein (cAMP production), Gαq-protein (intracellular Ca^2+^ mobilization) and β-arrestin (1 and 2) signalling were used to determine the potency (EC_50_; effector concentration for half-maximum response) and maximal efficacy (*E*_max_ (%)) of peptides relative to the reference ligand Ex4 (Table 1). Peptides P1, P2, P5 and P10 all stimulated cAMP production. However, only P5 functioned as a full agonist (*E*_max_=100%) displaying sub-nanomolar potency at both the human (hGLP-1R) and mouse receptor (mGLP-1R) (Fig. 2a,b; Table 1). The P5 EC_50_ was similar to the endogenous ligand GLP-1 but was slightly right shifted when compared with the reference peptide Ex4 (Fig. 2a,b; Table 1). Importantly, P5-induced cAMP production was inhibited by the selective GLP-1R antagonist Ex 9–39 in a concentration-dependent manner (Supplementary Fig. 1a,b). In addition, P5-induced cAMP production was negligible in HEK293 cells expressing the human glucagon receptor (Supplementary Fig. 1c). These data suggest that P5 selectively interacts with the GLP-1R.

We next investigated the ability of P5 to promote GLP-1R-mediated intracellular Ca^2+^ mobilization. P5 induced a concentration-dependent increase in [Ca^2+^]_*i*_ and displayed similar potency and efficacy when compared with the reference ligand Ex4 and (Fig. 2c; Table 1). When compared with GLP1, P5 was 3-folds more potent (Table 1). It is generally accepted that the GLP-1R-mediated increase in [Ca^2+^]_*i*_ in β-cells is due to GLP-1R activation of the Gαs pathway, inducing cAMP- and PLC/ Ca^2+^-dependent responses, rather than via Gαq*/*11 signal transduction. Nevertheless, the P5-induced increase in [Ca^2+^]_*i*_ in CHO-GLP-1R expressing cells was inhibited by the selective Gαq inhibitor FR900359 (refs 17, 18; Supplementary Fig. 1d) confirming that GLP-1R can functionally couple to Gαq.

Previous reports have demonstrated that the GLP-1R also directly couples to β-arrestin 1 and -2 following activation with Ex4 (refs 6, 7). Therefore we examined the ability of P5 to induce recruitment of β-arrestin 1 and -2 using a cellular assay that monitors the direct interaction between β-arrestin and the receptor. P5 showed a greatly diminished efficacy and potency (*E*_max_=29 and 32%; EC_50_=447 and 529 nM, for β-arrestin 1 and -2 recruitment, respectively), when compared with the reference agonist Ex4 (EC_50_=29.57 and 5.6 nM for β-arrestin 1 and -2 recruitment, respectively; Fig. 2d,e) and to the endogenous peptide GLP-1 (*E*_max_=71 and 77%; EC_50_=280 and 60 nM for β-arrestin 1 and -2 recruitment, respectively).

We further quantified the relative bias of P5 using an ‘equiactive' comparison and Ex4 as the reference ligand. The endogenous ligand GLP-1, did not demonstrate detectable bias, whereas P5 yielded bias factors (*β*), of 0.9 and 1.4 for Gαs-protein over β-arrestin 1 and -2, respectively, and of 1.7 and 2.4 for Gαq-protein over β-arrestin 1 and -2, respectively (Fig. 2f; Supplementary Table 1), thus confirming that P5 has a preference for promoting GLP-1R-mediated G-protein activation over β-arrestin recruitment (referred to as G-protein bias).

As β-arrestin recruitment is believed to play a key role in GPCR desensitization, we further investigated the effect of P5 and Ex4 on GLP-1R desensitization using a label-free assay which allows non-invasive bioimpedance-based measurement of an integrated cellular response^19^. In this assay, P5 and Ex4 induced concentration-dependent changes in cellular impedance and displayed similar potency (0.029 and 0.015 nM, respectively) and efficacy (Fig. 2g). It is well established that following a first stimulation, GPCRs that undergo desensitization display reduced responsiveness to a second stimulation with an agonist. Therefore, CHO-GLP-1R-expressing cells were stimulated for 60 min with 0.001, 0.01 or 0.1 nM of P5 or Ex4. Subsequently, the ligand-containing medium was removed; the cells were washed three times and further incubated in assay buffer for 30 min before being stimulated a second time with a fixed concentration of Ex4 (0.1 nM). In CHO-GLP-1R exposed to P5, the variation of cellular impedance induced by the second stimulation was similar to the control (Fig. 2h). In contrast, in CHO-GLP-1R exposed to Ex4 the response to a second stimulation was significantly attenuated in a concentration-dependent manner (Fig. 2h).These data suggest that P5 has less propensity to induce GLP-1R desensitization when compared with Ex4.

### P5 is a weak insulin secretagogue and improves glucose tolerance

**Figure 3:**
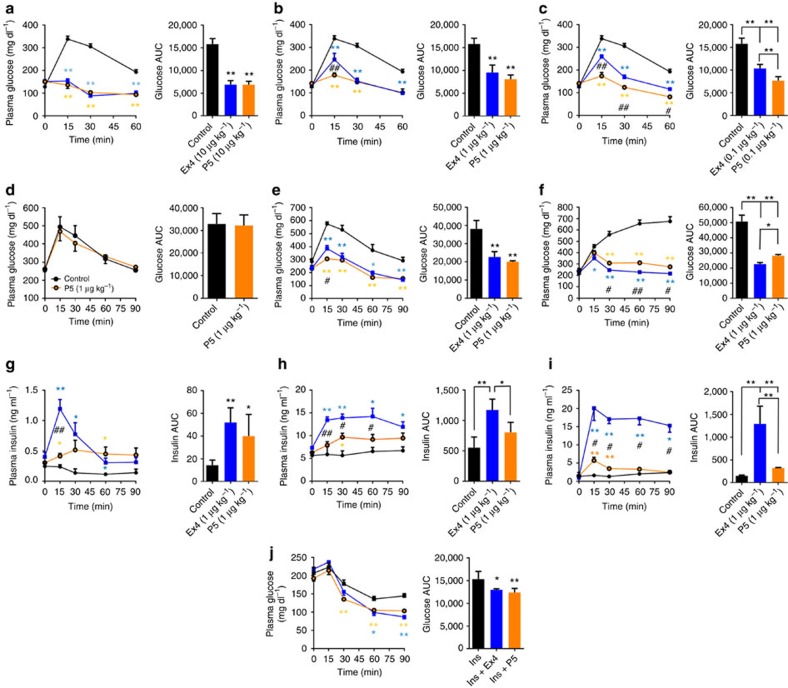
Acute administration of P5 lowers glucose in diabetic mice. Effect on intraperitoneal glucose tolerance after a single intraperitoneal co-injection of saline (black), Ex4 (blue) or P5 (orange) with glucose challenge (*n*=5). Glucose tolerance in lean mice treated with 10 μg kg^−1^ (**a**), 1 μg kg^−1^ (**b**) or 0.1 μg kg^−1^ (**c**), in GLP-1RKO treated with 1 μg kg^−1^ (**d**), in *ob/ob* mice treated with 1 μg kg^−1^ (**e**) and in DIO mice treated with 1 μg kg^−1^ (**f**). (**g**–**i**) Effect of P5 on plasma insulin levels. Plasma analysis was performed after a single intraperitoneal co-injection of saline (black), 1 μg kg^−1^ of Ex4 (blue) or 1 μg kg^−1^ of P5 (orange) with glucose challenge (*n*=5) in lean mice (**g**), in *ob/ob* mice (**h**) and in DIO mice (**i**). (**j**) Blood glucose levels measured in *ob/ob* mice after a single intraperitoneal co-injection of saline (black), Ex4 (blue) or P5 (orange) with insulin (*n*=5).Data are mean±s.e.m. Statistic by two-tailed *t*-test: *P<0.05; **P<0.01, comparing saline to peptide injection; #*P*<0.05; ##*P*<0.01, comparing Ex4 to P5 injection. AUC, area under the curve.

As P5 clearly demonstrated ligand bias *in vitro* with respect to Ex4, the challenge was to determine how the *in vitro* bias activity of P5 translates to *in vivo* efficacy. We compared the acute effects of escalating doses of P5 versus Ex4, on glucose tolerance, in lean mice; genetically induced obese mice (*ob/ob*); or diet-induced obese (DIO), insulin-resistant mice. In 8-week-old lean mice fed on normal chow, a single injection of P5 or Ex4 significantly improved glucose disposal in a glucose tolerance test (GTT) at doses ranging from 0.1 to 10 μg kg^−1^, when compared with injection of vehicle only (Fig. 3a–c). Interestingly, P5 dosed at 1 μg kg^−1^ lowered fasting blood glucose levels with a faster onset as indicated by a greater correction of glucose levels at the 15 min time point when compared with the same dose of Ex4 (178±10 versus 247±27 mg dl^−1^; Fig. 3b). Furthermore, at the lowest dose tested (0.1 μg kg^−1^), P5 showed improved efficacy at all time points and significantly decreased value of area under the curve (AUC; Fig. 3c). Collectively, these data indicate that P5 at 0.1 μg kg^−1^ improved blood glucose tolerance to the same extent as a 100-fold higher dose of Ex4. Furthermore, P5 failed to improve glucose tolerance in the GLP-1R knockout mice (GLP-1R^−/−^; Fig. 3d) indicating that the peptide does not display any apparent off-target effects that may contribute to its glucoregulatory activity. Together, these data indicate that in metabolically healthy lean mice, P5, a G-protein-biased agonist of the GLP-1R, has a sustained and greater glucoregulatory activity compared with the reference agonist Ex4.

We next compared the effect of escalating doses of P5 and Ex4 in 8-week-old leptin deficient (*ob/ob*) mice. Injection of P5 or Ex4 induced a reduction in blood glucose level in a dose-dependent manner (Fig. 3e; Supplementary Fig. 2a,b). In good agreement with our previous results, GTT of *ob/ob* mice treated with 10 μg kg^−1^ of P5 or Ex4 showed a similar and complete correction of glucose clearance in response to glucose challenge (Supplementary Fig. 2a). In this mouse model of T2DM, P5-treated animals also showed a slightly but significantly improved glucoregulatory activity at the 15 min time point compared with Ex4 when dosed at 1 μg kg^−1^ (Fig. 3e). In contrast to our previous observations in lean mice, *ob/ob* mice dosed with 0.1 μg kg^−1^ of P5 or Ex4 showed a similar correction of glucose clearance in response to a glucose challenge (Supplementary Fig. 2b). Nevertheless, both P5 and Ex4 significantly decreased the value of AUC of glucose tolerance test when compared with the control group. We next tested P5 efficacy in the DIO mouse model of pre-type 2 diabetes (obese hyperglycaemic and insulin-resistant mice). As previously reported^20^, DIO wild-type mice fed on the high-fat diet displayed impaired glucose tolerance (Fig. 3f) compared with wild-type mice fed with normal chow (Fig. 3a). A single injection of P5 or Ex4 (1 μg kg^−1^) significantly improved glucose clearance in response to the glucose challenge as indicated by a significant decrease in the AUC of GTT when compared with control; however, compared with Ex4, P5 was slightly less efficient in clearing glucose in the context of acute glycaemic control (Fig. 3f).

As GLP-1 agonists contribute to glycaemic control by potentiating glucose-dependent stimulation of insulin secretion from pancreatic β cells^21^, we compared the effects of the biased agonist P5 with Ex4 on plasma insulin levels in response to the glucose challenge. In chow-fed lean mice, P5 or Ex4 dosed at 1 μg kg^−1^ significantly increased the level of insulin compared with control mice (Fig. 3g). Surprisingly, although P5 displayed a greater glucoregulatory activity compared with Ex4 in lean mice (Fig. 3a–c), Ex4 potentiated the insulin secretory response to a greater extent than P5, reaching significantly higher plasma insulin level at the 15 min time point when compared with a similar dosing of P5 (Fig. 3g). This observation suggests uncoupling of the P5′s glucoregulatory activity from its insulin secretagogue activity. In hyperinsulinemic *ob/ob* mice, P5 dosed at 1 μg kg^−1^ only modestly potentiated the insulin secretory response when compared with control group, whereas Ex4 significantly increased the level of insulin when compared with either the control group or to the P5-treated group (Fig. 3h). At a higher dose (10 μg kg^−1^), P5 significantly increased plasma insulin level in *ob/ob* mice when compared with control group (Supplementary Fig. 2c). However, consistent with our previous observation, the insulin secretory response was significantly lower when compared with a similar dosing of Ex4 (Supplementary Fig. 2c). In DIO mice, a single injection of P5 or Ex4 (1 μg kg^−1^) significantly increased plasma insulin level when compared with control group (Fig. 3i). However, as we observed in lean chow-fed and *ob/ob* mice, plasma insulin levels were significantly lower in response to P5 compared with the same dose of Ex4. These data indicate that P5, a GLP-1R G-protein-biased agonist peptide, displays decreased insulin secretagogue activity relative to the reference agonist Ex4. Nevertheless, in an acute setting, P5 significantly improved glycaemic control in metabolically healthy animals as well as in both mouse models of T2DM suggesting that P5 might act as an insulin sensitizer. Therefore, we examined the effect of a single injection of P5 on insulin sensitivity using an insulin tolerance test in hyperinsulinemic *ob/ob* mice. Co-injection of P5 or Ex4 (1 μg kg^−1^) similarly enhanced the hypoglycaemia response to insulin (Fig. 3j) in an acute setting.

### P5 is more effective at controlling chronic hyperglycaemia

**Figure 4:**
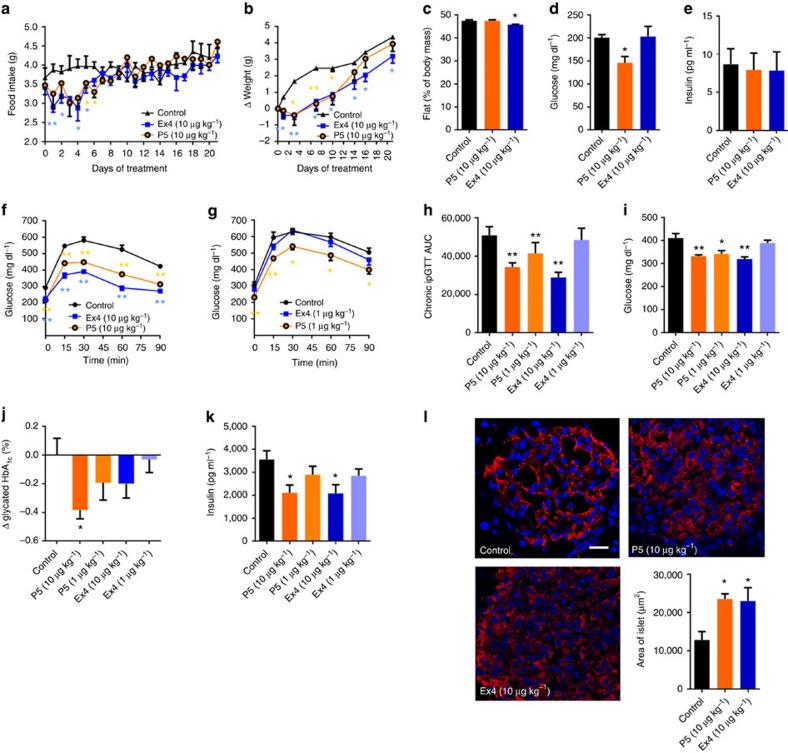
Chronic administration of P5 improves glycaemic status in diabetic mice. Effect on food intake (**a**), body weight (**b**), fat mass (**c**), *ad libitum*-fed plasma glucose (**d**) and insulin (**e**) levels following daily subcutaneous injection of saline (black), Ex4 (blue) and P5 (orange) at 10 μg kg^−1^ in *ob/ob* mice. (**f**–**h**) Effect of 10 μg kg^−1^ (**f**,**h**; darker shade) and 1 μg kg^−1^ (**g**,**h**; lighter shade) of P5 or Ex4 on intraperitoneal glucose tolerance following daily subcutaneous injection of saline (black), Ex4 (blue) and P5 (orange) in DIO mice. (*n*=8). Effect of P5 or Ex4 on *ad libitum*-fed plasma glucose level (**i**), HbA_1C_ (**j**), insulin plasma level (**k**), islet morphology (**l**) after daily subcutaneous injections of saline (black), Ex4 (blue) and P5 (orange) at 1 μg kg^−1^ (lighter shade) or 10 μg kg^−1^ (darker shade) (*n*=6–8). Scale bar, 20 μm. Data are mean±s.e.m. Statistic by two-tailed *t*-test: *P<0.05; **P<0.01, comparing saline to peptide injection. AUC, area under the curve.

To determine whether chronic treatment with the GLP-1R G-protein-biased agonist can improve hyperglycaemia, *ob/ob* mice were treated daily with 10 μg kg^−1^ of P5 or Ex4 for 3 weeks. Both peptides significantly decreased food intake (Fig. 4a) and body weight (Fig. 4b) during the initial phase of treatment. Nevertheless, by day 21 of treatment, in contrast to Ex4, P5 did not significantly affect body weight or fat mass when compared with vehicle-treated *ob/ob* mice (Fig. 4b,c). However, treatment with P5 significantly decreased *ad libitum*-fed blood glucose levels (Fig. 4d) without affecting insulin levels (Fig. 4e) whereas Ex4 treatment had no effect on glycaemia (Fig. 4d). Importantly, the differences in Ex4 and P5 *in vivo* efficacy are not due to differences in peptide pharmacokinetics as both peptides display similar half-life in mouse serum (supplementary Fig. 3).These data suggest that P5 has superior antihyperglycaemic efficacy in diabetic *ob/ob* mice.

We further determined whether similar metabolic improvement can be obtained in a mouse model of insulin resistance. Therefore DIO mice were treated daily with escalating doses of P5 or equimolar doses of Ex4 for 4 weeks. When dosed at 10 μg kg^−1^, both P5 and Ex4 significantly lowered fasting blood glucose levels (∼25%; Fig. 4f) and improved glucose tolerance (Fig. 4f,h) compared with vehicle-treated animals. At the lowest dose tested (1 μg kg^−1^), P5 significantly decreased fasting blood glucose levels and significantly attenuated glucose intolerance whereas an equivalent dose of Ex4 had no effect (Fig. 4g,h). Furthermore, the GLP-1R G-protein-biased agonist dosed at 1 or 10 μg kg^−1^ significantly decreased *ad libitum*-fed blood glucose levels when compared with vehicle control whereas Ex4 showed efficacy only at the highest dose tested (Fig. 4i). We next determined the haemoglobin (HbA_1c_) levels in DIO mice treated for 4 weeks with P5 and Ex4 as an index of long-term blood glucose regulation. P5, at the highest dose tested, produced a significant decrease in HbA_1c_ when compared with control whereas chronic treatment with Ex4 only modestly decreased HbA_1c_ levels (Fig. 4j). The mean change in HbA_1c_ ranged from −0.19% to −0.38% in DIO mice treated with P5 at 1 and 10 μg kg^−1^, respectively. The superior effect of P5 in lowering HbA_1c_ level correlates well with its superior efficacy in lowering both fasting and non-fasting blood glucose level at 1, and 10 μg kg^−1^. Notably, both peptides significantly decreased the concentration of circulating insulin and C-peptide at the highest dose (Fig. 4k; Supplementary Fig. 4a). In addition, histological analyses of the pancreas revealed that both the G-protein-biased agonist and Ex4 preserved proper insulin immunoreactivity (Fig. 4l, red), and significantly increased islet area (Fig. 4l). Together these data indicate that P5 has superior antihyperglycaemic efficacy compared with Ex4 in hyperglycaemic *ob/ob* mice as well as in insulin-resistant DIO mice.

**Figure 5:**
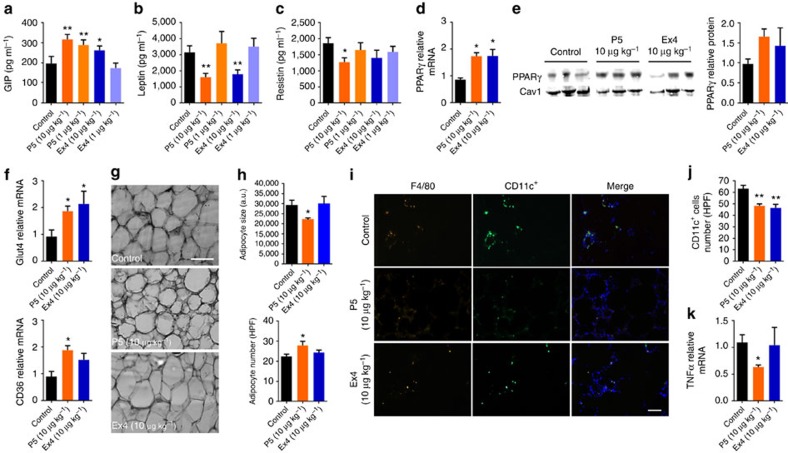
Chronic administration of the G-protein-biased agonist modulates adipogenesis and insulin sensitivity in DIO mice. Effect of P5 or Ex4 on GIP (**a**), leptin (**b**) and resistin (**c**) plasma levels following daily subcutaneous injections of saline (control), Ex4 or P5 (*n*=6). (**d**) Real-time quantitative PCR (qPCR) analysis on the expression of the gene encoding PPARγ (*n*=6) and (**e**) western blot analysis of PPARγ protein expression in epididymal white adipose tissue (eWAT) following daily subcutaneous injections of saline (control), Ex4 or P5. Caveolin-1 (Cav1) was used as loading control. (**f**) qPCR analysis of eWAT mRNA expression of genes regulated by PPARγ activity Glut4 and CD36 following daily subcutaneous injections of saline (control), Ex4 or P5 (*n*=6). (**g**) Representative microscopy images of eWAT from DIO mice following daily subcutaneous injections of saline (control), Ex4 or P5. Sections were stained using hematoxylin and eosin (*n*=4). Scale bar, 100 μm. (**h**) Adipocyte cell size and number per high-powered field (HPF) in DIO mice following daily subcutaneous injections of saline (control), Ex4 or P5. (**i**) Representative immunofluorescence microscopy images of eWAT co-stained with F4/80 and CD11c. Scale bar, 50 μm. (**j**) Quantification of F4/80^+^ CD11c^+^ cells per high-powered field (HPF) in DIO mice following daily subcutaneous injections of saline (control), Ex4 or P5 (*n*=4). (**k**) qPCR analysis of eWAT mRNA expression of tumour-necrosis factor-α in DIO mice following daily subcutaneous injections of saline (control), Ex4 or P5 (*n*=6). Data are mean±s.e.m. Statistic by two-tailed *t*-test: *P<0.05; **P<0.01, comparing saline to peptide injection.

As the G-protein-biased agonist is a weak insulin secretagogue, it is likely that in the context of chronic treatment additional β-cell-independent mechanisms contribute to its superior glucoregulatory activity. Therefore, we further investigated the effects of P5 on the circulating levels of glucagon and glucose-dependent insulinotropic polypeptide (GIP), two other hormones involved in the regulation of glucose homeostasis. At the highest dose tested, both peptides tend to decrease glucagon level (Supplementary Fig. 4b), but interestingly, treatment with both 1 and 10 μg kg^−1^ of P5 resulted in a significant increase in GIP level whereas Ex4 showed efficacy only at the highest dose (Fig. 5a). In addition, we monitored body weight, and determined the effect of chronic treatment with P5 or Ex4 on the levels of adipose tissue-secreted hormones including leptin and resistin in DIO mice. Consistent with our previous observation in *ob/ob* mice, at the highest dose tested (10 μg kg^−1^), both P5 and Ex4 induced a modest decrease in body weight during the initial phase of treatment (Supplementary Fig. 5a,b). However, in contrast to Ex4, P5 dosed at 10 μg kg^−1^ did not decrease fat mass when compared with vehicle-treated animals (Supplementary Fig. 5c). Nevertheless, at the highest dose tested P5 was capable of decreasing circulating leptin levels similarly to Ex4 (Fig. 5b). Notably, chronic treatment with 10 μg kg^−1^ of P5 resulted in a significant decrease in circulating levels of resistin (Fig. 5c), an adipokine shown to negatively impact glucose metabolism and insulin sensitivity^22^. In contrast, Ex4 had no significant effect on resistin levels (Fig. 5c). Because leptin and resistin levels are regulated by the activity of peroxisome proliferator-activated receptor gamma (PPARγ) in epididymal white adipose tissue (eWAT), we further examined the expression of PPARγ^23^,^24^. PPARγ mRNA expression was significantly upregulated and protein levels were nearly 1.7-fold greater in mice treated with P5 or Ex4 when compared with vehicle-treated DIO mice (Fig. 5d,e). Consistently, both treatments also lead to an upregulation of the PPARγ-regulated gene Glut4, a protein implicated in glucose uptake in response to insulin (fig. 5f). Interestingly, the PPARγ-regulated gene CD36 was significantly upregulated only in eWAT from P5-treated DIO mice (Fig. 5f) suggesting that P5 and Ex4 treatments might differentially affect PPARγ activity. As PPARγ and CD36 are involved in adipocyte differentiation and adipogenesis we performed histological analyses of eWAT^25^,^26^. DIO mice treated with 10 μg kg^−1^ of P5 had smaller and more numerous adipocytes when compared with control or Ex4-treated animals (Fig. 5g,h). These data suggest that in DIO mice treated with the G-protein-biased agonist, the adipose tissue mass increases via hyperplasia (increasing cell number) rather than via hypertrophy (increasing adipocyte size). Previous studies demonstrated that in mice on high-fat diet, eWAT expansion correlates with the accumulation of pro-inflammatory macrophages into adipose tissue contributing to insulin resistance^27^,^28^. Nevertheless, P5 significantly reduced the number of pro-inflammatory F4/80^+^-CD11c^+^ macrophages (Fig. 5i,j) and the expression of the cytokine tumour-necrosis factor-α in eWAT (Fig. 5k). Together, these data suggest that P5 and Ex4 induce distinct changes in eWAT consistent with improved insulin responsiveness.

**Figure 6:**
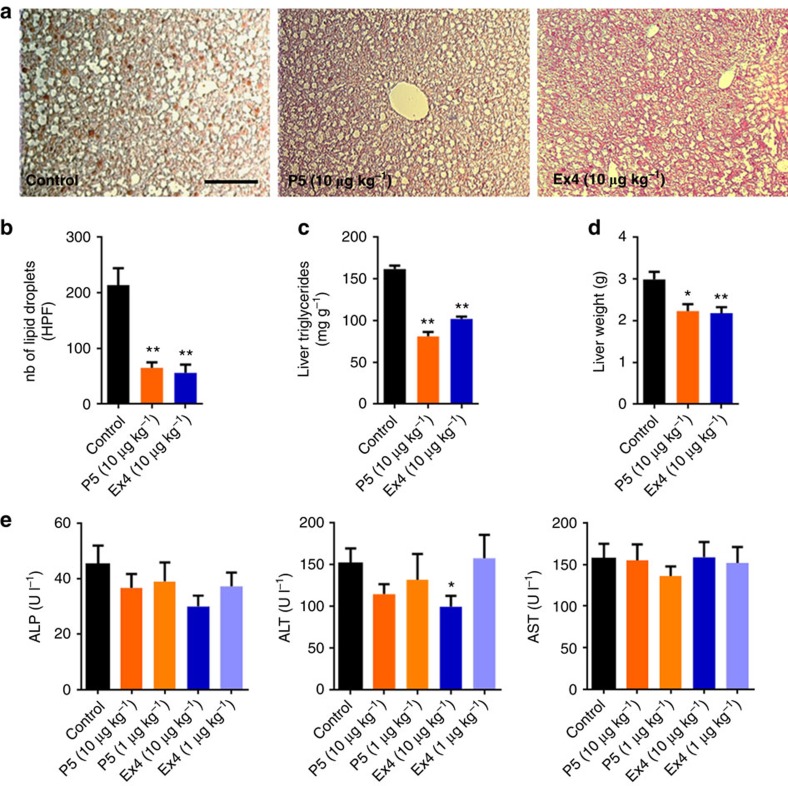
Chronic administration of the G-protein-biased agonist improves hepatic steatosis in DIO mice. (**a**) Representative microscopy images of liver tissue from DIO mice following daily subcutaneous injections of saline (control), Ex4 or P5 and (**b**) lipid droplet number per HPF. Section were stained with Oil Red O to investigate the presence of lipids in the liver (*n*=4). Scale bar, 100 μm. (**c**) Liver triglyceride content normalized with liver weight and (**d**) liver weight in DIO mice following daily subcutaneous injections of saline (control), Ex4 or P5. (**e**) Effect of P5 or Ex4 on plasma on alkaline phosphatase (ALP), alanine aminotransferase (ALT) and aspartate aminotransferase (AST) (*n*=8). Data are mean±s.e.m. Statistic by two-tailed *t*-test: *P<0.05; **P<0.01, comparing saline to peptide injection.

Chronic treatment with P5 and Ex4 significantly reduced ectopic lipid deposition in the liver as indicated by a decrease in the number of lipid droplets (Fig. 6a,b), reduced triglycerides levels (Fig. 6c) and liver weight (Fig. 6d). The G-protein-biased agonist performed equally well to reduce circulating low-density lipoprotein levels without affecting high-density lipoprotein or triglycerides levels when compared with similar dosing of Ex4 (Supplementary Table 2). Importantly, chronic treatment with P5 did not induce hepatocellular toxicity as reflected by the absence of changes in plasma level of alanine aminotransferase, alkaline phosphatase and aspartate aminotransferase (Fig. 6e). Collectively, these data suggest that chronic treatment with a GLP-1R G-protein-biased agonist peptide maintains eWAT mass by increasing adipogenesis and prevents ectopic fat deposition in the liver induced by high-fat diet.

## Discussion

GPCR peptide ligands have always been noted for their selectivity, potency and rapid optimization, however, typically peptides are chemically synthesized which is a low-throughput process that intrinsically lacks diversity. Although affinity-based screening technologies such as phage and yeast display enable the high-throughput screening of large combinatorial peptide libraries, they rely on the use of recombinant protein or membrane preparations and do not distinguish between agonists, inverse agonists and antagonists. To address this, we have developed a functional cell-based, autocrine system for the selection of ligands from large intracellular combinatorial peptide libraries. This functional cell-based system, can allow screening of 100 million peptides at 2 million events per hour. In the proof-of-concept experiments presented here, we have generated and screened three distinct peptide libraries and show how this methodology allows one to detect and identify novel GLP-1R ligands with unique pharmacology. Moreover, this autocrine-based selection system is a powerful approach that can be used to deorphanize orphan GPCRs and be applied to other cell surface receptors.

GLP-1R agonist-based therapies have emerged as a pharmaceutical approach for the treatment of T2DM. Although the currently approved treatments with GLP-1R agonists demonstrate remarkable antidiabetic effects, further improvements in this class are expected such as long-term glucose control^29^. In addition, adverse side effects and concerns regarding safety profile following prolonged use of GLP-1R-based therapeutics have emerged^30^. Optimization of GLP-1R agonist therapy often focuses on increasing the plasma half-life of GLP-1 and GLP-1 mimetics. Recently, the concept of ‘ligand bias' has provided new avenues for the development of drugs with novel modes of action that have the potential to provide greater therapeutic value while possibly avoiding unwanted side effects^13^. In response to GLP-1 or Ex4, the GLP-1R signals through G-protein-regulated pathways as well as through G-protein-independent pathways by engaging the scaffold protein β-arrestin. Despite major advances in understanding GLP-1R function, a better understanding in the relative implication of these distinct pathways in physiological functions may help the development of improved therapeutics. Here we identified a potent and selective GLP-1R G-protein-biased agonist namely P5, and explored its efficacy in mouse models of T2DM. A direct comparison of P5 with the reference peptide Ex4 revealed that G-protein signalling downstream of GLP-1R activation in the absence of β-arrestin signalling is sufficient to correct hyperglycaemia, improve insulin sensitivity, preserve pancreatic islet integrity and improve liver steatosis. Nevertheless, although P5 and Ex4 treatment result in some comparable physiologic responses, our data demonstrate differences in the G-protein-biased agonist mode of action and highlights the divergent roles of GLP-1R-mediated β-arrestin signalling in the β-cell and adipocyte. In the β-cell, loss of β-arrestin signalling resulted in impaired insulin secretion, whereas it promoted hyperplasia in eWAT and reduced plasma adipokine levels.

To enable the discovery of novel GLP-1R G-protein-biased agonists displaying selectivity and high potency for the GLP-1R, combinatorial peptide libraries were designed based on the competitive GLP-1R antagonist Ex 9–39 sequence to which 7–10 random amino acids have been added to the N terminus. These libraries were screened in a high-throughput, autocrine manner using a CRE-driven GFP reporter system. Extensive *in vitro* pharmacological characterization of the active peptides revealed that P5 displayed comparable efficacy to the endogenous ligand GLP-1 and its analogue Ex4 with regards to G-protein signalling. However, in contrast to GLP-1 and Ex4, P5 exhibits a severely blunted β-arrestin response. Oxyntomodulin, another endogenous ligand of the GLP-1R and the glucagon receptor and allosteric ligands of the GLP-1R have been reported to exhibit some degrees of G-protein bias at the GLP-1R^31^,^32^. Nevertherless, in contrast to these molecules, P5 displayed high potency at the GLP-1R and was devoid of activity at glucagon receptor. Consequently, P5 represents the only selective and potent GLP-1R G-protein-biased orthosteric agonist. Interestingly, examination of the peptide sequence revealed that P5 N-terminal residues are distinct from residues 1–8 of the endogenous hormone or Ex4 suggesting that extensive sequence modifications rather than minimal structural changes (single amino acid change) are required to produce biased agonism at the GLP-1R. These observations clearly demonstrate that the autocrine-based screening method is a powerful tool enabling the identification of novel peptides displaying new pharmacological virtues. In addition, the superior glucoregulatory activity achieved with P5 chronic treatment suggests that this novel sequence retains the resistance to dipeptidyl peptidase-IV (DPP-IV)-mediated degradation as exhibited by Ex4.

In an acute setting, this G-protein-biased agonist significantly improved glycaemic control in metabolically healthy animals as well as in mouse models of T2DM. In metabolically healthy mice P5 improved blood glucose tolerance to the same extent as a 100-fold higher dose of Ex4. These observations along with the pharmacokinetics study suggest that the difference in P5 efficacy is inherent to the peptide. In this reductionist approach, P5 significantly increased insulin level when compared with control. However, the P5 secretagogue activity was significantly decreased relative to the reference peptide Ex4. These results suggest that *in vivo,* β-arrestin 1/2 signalling downstream of GLP-1R activation plays a critical role in potentiating glucose-stimulated insulin secretion. These data are consistent with previous reports demonstrating that β-arrestin 1/2 are indispensable for the potentiating action of GLP-1 on glucose-stimulated insulin secretion in cultured pancreatic β cells^6^,^7^.

Chronic treatment with the G-protein-biased agonist resulted in enhanced correction of hyperglycaemia in the hyperglycaemic *ob/ob* mice as well as in the insulin-resistant DIO mice when compared with the reference agonist Ex4, as reflected by a significant decrease in blood glucose and HbA1c levels. P5 increased glucoregulatory efficacy could not be attributed to an enhanced insulinotropic effect, as P5 secretagogue activity was significantly decreased compared with Ex4 suggesting that additional and distinct mechanisms might contribute to improve glycaemia. In this view, we found that chronic treatment with P5-induced changes in gene expression that are consistent with improved insulin sensitivity. In eWAT, PPARγ expression and activity were increased leading to changes in PPARγ-regulated genes including Glut4 (ref. 33), CD36 (ref. 34) and tumour-necrosis factor-α (ref. 35). Moreover, consistent with the increase in PPARγ and CD36 expression^25^,^26^, P5 chronic treatment significantly increased the number of small adipocytes in eWAT without decreasing net body fat suggesting that the G-protein-biased agonist promotes *de novo* differentiation of preadipocytes which reside in the stromal vascular fraction of adipose tissue. At the same time, P5 prevented the accumulation of macrophages in eWAT contributing to decrease inflammation and insulin resistance^26^. As smaller adipocytes are highly insulin sensitive^36^, P5 by shifting the balance towards hyperplasia might restore insulin signalling and increase lipid storage capacity in eWAT resulting in improved glucose homeostasis and redistribution of lipids from ectopic deposits in liver to adipocytes. In addition, chronic treatment with the G-protein-biased agonist decreased circulating levels of insulin, and adipokines including leptin and resistin. Although the roles of resistin, a hormone produced and secreted from mature adipocytes and macrophages^37^,^38^, may vary between physiological and pathophysiological states and among species^39^, several reports suggest that in mice, resistin directly affects glucose metabolism by antagonizing insulin action^40^,^41^. The molecular mechanisms underlying these changes in eWAT are complex but are likely related to the substantial increase in PPARγ levels in adipocytes as previously reported^42^. However, further studies are warranted to determine how chronic treatment with P5 affects PPARγ activity and systemic insulin sensitivity in DIO mice.

In line with previous reports^43^,^44^,^45^ our data support the notion that non β-cell actions of GLP-1 agonists can improve glycaemic control. Importantly, GLP-1R is expressed in adipose tissue, in both the stromal vascular and the adipocyte fraction and its expression level has been found to correlate with the degree of insulin resistance^46^. In addition, the GLP-1 peptide has been reported to regulate adipogenesis *in vitro*^47^,^48^. Given that P5, a G-protein-biased agonist with a severely blunted β-arrestin response has less propensity to induce GLP-1R desensitization, sustained activation of the receptor in adipose tissue may lead to the changes we observed in eWAT. Consistent with this notion, increased expression of adipogenic genes and a decrease in resistin expression was reported in β-arrestin 1 knockout mice^49^. Nevertheless, considering the multitude of metabolic pathways regulated by β-arrestin, further studies are warranted to determine the role of β-arrestin signalling downstream of GLP-1R activation in adipogenesis. Additionally, we found that chronic treatment with P5 increased circulating level of GIP to a greater extent than Ex4. Several studies demonstrated that GIP acts as an insulin sensitizer in adipocytes and disruption of the GIP/GIP-R axis has been reported in insulin-resistant states such as obesity^50^,^51^. Interestingly, PPARγ activation was shown to increase GIP-R levels during adipocyte differentiation^52^. Thus, by increasing GIP and PPARγ levels, P5 chronic treatment may restore GIP/GIP-R signalling in adipocytes. Furthermore, previous studies have demonstrated that the simultaneous activation of the GLP-1R and the GIP-R results in enhanced glycaemic control, and lower HbA1c levels in human and rat, when compared with GLP-1R alone^53^, suggesting a GIP and GLP-1 synergism. Thus, the superior glycaemic control observed with the G-protein-biased agonist may result from P5-induced increases in GIP level and concomitant receptor activation. In addition, the GLP-1R can form homodimers as well as ligand-induced heterodimers with the GIP-R^54^. It is conceivable, that P5 may promote the formation of new and pharmacologically distinct homo/heterodimers displaying different signalling capacity. However, further studies are required to delineate more precisely the molecular and cellular mechanisms and the consequences of P5-induced increase in GIP levels.

**Figure 7:**
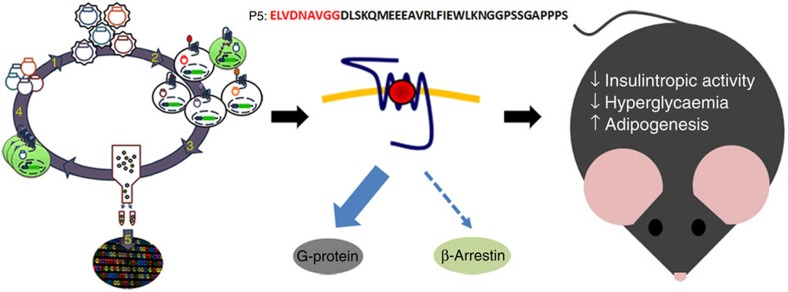
Schematic depicting the identification and characterization of a novel GLP-1R-biased agonist. Using an autocrine-based system coupled to FACS, we screened large, diverse, combinatorial peptide libraries and identified P5, a potent and selective G-protein-biased GLP-1R agonist. P5 displayed a decreased insulinotropic effect, yet significantly improved glucose tolerance and insulin responsiveness by promoting white adipocyte tissue hyperplasia.

In summary, high-throughput autocrine-based functional screening of combinatorial peptide libraries enabled the discovery of a high potency G-protein-biased GLP-1R agonist demonstrating new pharmacological virtues. In a series of translational preclinical studies we demonstrate that P5 is a weak insulin secretagogue yet displays superior antidiabetic effect (Fig. 7). Thus, GLP-1R G-protein-biased ligands may offer new and unappreciated advantages in the context of chronic treatment such as promoting adipocyte hyperplasia, restoring insulin responsiveness and long-term glycaemic control while preserving pancreatic β-cell function by minimizing the insulin secretory burden.

## Methods

### Construction of an Exendin-4-based membrane-tethered combinatorial peptide library

The expression of transmembrane-tethered Exendin-4 was under control of EF1a in the lentiviral vector. The arrangement of genes are in the order of: interleukin 2 signal peptide at the N terminus; Exendin-4; tandem repeats of the GGGGS linker; a PDGFR transmembrane region; and mCherry fused at the intracellular side of the PDGFR transmembrane region. To construct the combinatorial peptide libraries, the Exendin 9–39 (Ex 9–39) antagonist as anchor site to GLP-1R extracellular domain was kept constant. Randomized peptides in the format of *X*_7_, C*X*_5_C*X*_2_ and C*X*_4_C*X*_3_ (*X*= 20 natural amino acids) were appended to the N terminus of Ex 9–39 by PCR using oligonucleotides with degenerate codons. The diversity of each library is ∼300 thousand members. The lentiviral library was prepared by co-transfection of HEK293T cells with the library plasmid and the package plasmid. Supernatants containing virus were collected at 48 h post transfection. The titre of lentivirus preparations was determined using Lenti-X p24 ELISAs (Clontech).

### FACS-based sorting

HEK293-GLP-1R-GFP cells were transduced with the lentivirus peptide library. Two days post-infection GFP-positive cells were sorted using a MoFlo Astrios fluorescence-activated cell sorter (Beckman Coulter's). The peptide sequences were recovered directly from sorted cells by PCR and cloned into lentiviral vectors to construct libraries for next round selection. Lentivirus were prepared and HEK293-GLP-1R-GFP reporter cells were transduced for the next round of sorting. Three Iterative rounds of selection were carried out.

### Sequencing and bioinformatics analysis

Sequencing was performed on the Ion PGM System with an Ion PGM Sequencing 400 Kit and a 318 v2 chip for a total of 850 nucleotide flows. The standard base calling procedure was used in raw data processing. A total of 7,042,611 reads were generated with a mean read length of 489 bp. An in-house bioinformatics pipeline was developed to process and analyse the deep sequencing data of peptide libraries generated by PGM. The pipeline processing consists of four steps: each sequence read will be: (1) reformed and labelled with a unique index number; (2) aligned to the N- and C-terminal flanking regions to extract the peptide sequence; (3) compared with the template peptide sequences at the nucleotide level using a global alignment module in CLUSTALW2; and (4) translated to amino acid sequences to determine whether there are errors in the peptide region. The filtered and annotated peptide libraries were used for frequency analysis and clustering analysis.

### Peptide synthesis and purification

Rink Amide ChemMatrix resin was swelled overnight in dimethylfomamide (DMF). Peptide synthesis was carried out following standard Fmoc solid-phase peptide synthesis (SPPS) using 5 equiv. of Fmoc protected amino acid, 5 equiv. of HCTU (0.4 M in DMF) and 7.5 equiv. of DIEA for each coupling (20 min) and 20% 4-Me-piperidine (2 × 2 min) treatments for Fmoc deprotection. The peptide was cleaved from the resin using a standard TFA cleavage cocktail (95% TFA, 2.5% TIPS, 2.5% H_2_O) for 2 h, filtered, precipitated with cold diethyl ether and lyophilized from buffer (0.05% TFA in water), yielding the peptide as the TFA salt. Following purification by preparative reversed phase (high-performance liquid chromatography), a sample was injected on analytical HPLC to determine a final purity of 97.7% by HPLC peak area. Reconstructed ESI-MS data for the purified peptide is shown Supplementary Fig. 6 (calculated mass=4,224.6 Da, observed mass=4,224.0 Da).

### Cell culture

HEK293-GLP-1R-GFP cells (generated in-house using HEK293 cell line purchased from ATCC) were cultured in Dulbecco's modified Eagle's medium (DMEM) supplemented with 10% fetal bovine serum, 1 mg ml^−1^ geneticin and 100 μg ml^−1^ hygromycin. The stable CHO cell line (generated in-house using CHO-K1 cell line purchased from ATCC) expressing a functional human GLP-1R was cultured in F12 medium (Gibco) supplemented with 10% fetal bovine serum and 500 μg ml^−1^ geneticin. The HEK293 cell line expressing a functional mouse GLP-1R and the HEK293 cell line (generated in-house using HEK293 cell line purchased from ATCC) expressing a functional human glucagon receptor were cultured in DMEM supplemented with 10% fetal bovine serum and 10 μg ml^−1^ blasticidin. PathHunter EA-β-Arrestin 1 CHO cells and PathHunter EA-β-Arrestin 2 CHO cells expressing the human GLP-1R (DiscoveRx) were cultured in F12 medium supplemented with 10% fetal bovine serum, 1 mg ml^−1^ geneticin and 100 μg ml^−1^ hygromycin.

### *In vitro* pharmacology

cAMP HTRF assay to measure the effects of peptide-induced GLP-1R-mediated stimulation of cAMP production was performed according to manufacturer's instruction (cAMP dynamic 2 HTRF assay kit, Cisbio). Briefly, cells were plated in 20 μl growth medium at 1,000 cells per well in a white 384-well plate and cultured overnight. The following day, 5 μl of peptides prepared as 5 × solution in phosphate buffer saline (PBS) were added to the cells at the indicated concentrations. When GLP-1 was tested PBS was supplemented with DPP IV inhibitor (Tocris). Following 5 min incubation at room temperature, the cells were lysed in the detergent buffer containing the HTRF conjugates. The amount of cAMP in lysate samples was quantified on the Envision plate reader (PerkinElmer). To measure the effects of peptide on intracellular calcium mobilization, CHO cells stably expressing the human GLP-1R were seeded into black-walled 384-well plates at a density of 10,000 cells per well in 20 μl of growth medium and cultured overnight. The following day medium was removed and replaced with 20 μl of loading medium consisting of 1:1 (v/v) Opti-MEM: Hanks' balanced salt solution (HBSS), 2.5% (v/v) FBS, 20 mM HEPES, pH 7.4, 2.5 mM probenicid and fluorescent indicator 2 μM Fluo-4 AM (Invitrogen). Following a 60 min incubation of cells in the loading medium, cell and compound plates were placed into the FLIPR (Molecular Devices). Peptides (prepared as 5 × solution in HBSS and 20 mM HEPES, pH 7.4) were added at time=10 s and changes in fluorescence were monitored over a period of 250 s following excitation at a wavelength of 488 nm and detection at 510–560 nm. Relative changes over baseline (Δ*F*/*F*) were determined. To test for Gq pathway signalling, 1 μM of FR900359 (UBO-QIC, University of Bonn) was added to the loading medium. The PathHunter β-arrestin recruitment assays to measure the effects of the peptide on β-arrestin recruitment were performed according to the technical manual's instruction (PathHunter β-arrestin recruitment assay, DiscoveRx). Briefly, PathHunter EA-β-Arrestin CHO cells expressing the GLP-1R (DiscoveRx) were seeded overnight in white 384-well plates at 10,000 cells per well in 20 μl of F12 medium and incubated overnight. The following day, the medium was removed and replaced with 20 μl of Opti-MEM (Gibco). Cells were then stimulated with peptides (prepared as 5 × solution in PBS) or vehicle for 90 min at room temperature. Detection reagent was added, and luminescence was read on the Envision plate reader. For all the cell-based assays, concentration–response curves were recorded with four wells per concentration and experiment. Impedance measurements were performed using the Cellkey system (Molecular Devices). CHO-GLP-1R cells were seeded at a density of 45,000 cells per well into CellKey microplates (MDS SCIEX) in 100 μl of growth medium and cultured overnight. The following day, cells were washed three times with assay buffer (HBSS containing 20 mM HEPES, pH 7.4) and allowed to equilibrate for 30 min. After a 90 s read to establish a baseline, ligands (5 × in assay buffer) were added online in the CellKey instrument and the changes of cellular impedance were measured over 60 min. Subsequently, the cells were washed three times with assay buffer to remove the ligand and allowed to equilibrate in assay buffer for 30 min before being stimulated a second time with a fixed concentration of Ex4 at an EC_90_ (5 × in assay buffer). The changes of cellular impedance induced by a second addition were measured over 10 min. Six wells per concentration and experiment were recorded. The effects of peptides were calculated relative to the stimulation obtained with a maximally active concentration of Ex4. Concentration–response curves were determined by nonlinear regression analysis using Prism software (GraphPad Software Inc.). Ligand biased was determined using the equiactive comparison as previously described^55^. A bias factor (*β*), which quantifies the relative engagement of one signalling state over another compared with the reference peptide, was calculated using the following equation:


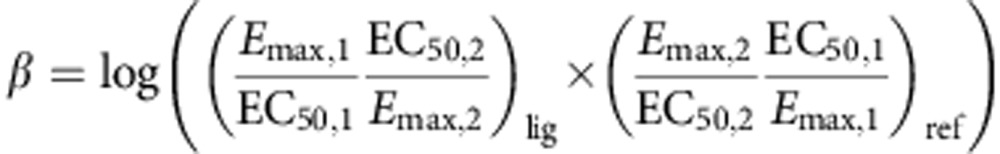


Where *E*_max_ is the maximal effect, EC_50_ is the half maximal concentration of the peptide, *lig* is the peptide being tested (P5 or GLP-1); *ref* is the reference peptide (Ex4); 1 is for values for the G-protein pathway; and 2 for the β-arrestin pathway.

### Animals

Mice were group housed on a 12:12 light–dark cycle in a temperature-controlled environment with free access to food and water. Male C57BL/6 mice (Jackson Laboratories) and male *ob/ob* mice (B6.Cg-Lep^ob^; Jackson Laboratories) were fed standard chow diet (Tekland Global Diet 2920X; Harlan). The mice were between the ages of 8 and 10 weeks when used in the studies. Male DIO mice, (C57BL/6 mice; Jackson Laboratories) were fed a diabetogenic diet, which is high-fat diet with 60% kcal from fat (high-fat diet (60%) diet D12492; Research Diets), for a minimum of 18 weeks before initiation of the studies and were between the ages of 6 and 7 months. GLP-1RKO mice (Dr D. Drucker, General Hospital, Ontario) were backcrossed onto the C57BL/6 genetic background and wild-type littermate were bred in-house and fed standard chow diet. All mice studies were approved by and performed according to the guidelines of the Institutional Animal Care and Use Committee of the Scripps Research Institute.

### Food-intake monitoring

Continuous monitoring of food intake was performed using the BioDAQ monitoring system. Recordings were halted for ∼1 h per day for animal maintenance and dosing, during which animals did not have access to food or water. Data were recorded using BioDAQ monitoring software 2.1.00, and analysed using DataViewer 2.2.02 and Prism software.

### Body composition measurement

Whole-body composition was measured with nuclear magnetic resonance technology (Minispec LF-50/mq 7.5 NMR; Brucker Optics).

### Blood parameters

Blood was collected from tail veins or after euthanasia, using heparinized microhematocrit tubes (SafeCrit) or EDTA-coated microcuvette tubes (Sarsdedt), respectively, centrifuged at 5,000*g* at 4 °C for 5 min and plasma was stored at −80 °C. Plasma insulin was quantified using AlphaLISA Insulin kit (PerkinElmer) and the Mouse Metabolic Magnetic Bead Panel (Millipore). Plasma c-peptide, glucagon and GIP were measured using the Mouse Metabolic Magnetic Bead Panel. Plasma triglyceride, high-density lipoprotein, low-density lipoprotein, alkaline phosphatase, alanine aminotransferase and aspartate aminotransferase levels were measured by enzymatic assays kits (Roche). HbA1C values were determined using A1CNow kit (Bayer). All assays were performed according to the manufacturer's instructions.

### Glucose and insulin tolerance test

GTTs and insulin tolerance tests were conducted after fasting, the C57BL/6 mice and the GLP-1RKO mice for 4 h and the *ob/ob* mice and the DIO mice for 8 h. Mice were injected intraperitoneal with 2 g of glucose per kg body weight or with insulin (0.75 U kg^−1^) and tail blood glucose level was monitored at the indicated time points using an AphaTRAK2 glucose metre (Abbott).

### Pharmacokinetics

Eight-weeks-old male C57BL/6 mice (*n*=3 per group) were injected with a single subcutaneous dose of P5 or Ex4 (100 μg kg^−1^), and blood was collected 1, 2, 4, 8 and 24 h later. Plasma concentration of P5 and Ex4 were determined using the Exendin-4 enzyme immunoassay (EIA) Kit (Phoenix Pharmaceutical) which recognizes Ex 9–39.

### Immunoblotting

Epididymal white adipose tissue were lysed in RIPA lysis buffer containing complete protease inhibitors (Roche Diagnostic), sonicated and centrifuged at 14,000 g for 15 min at 4 °C. Protein concentrations of the lysates were measured using the BCA Protein Assay Reagent (Pierce). 20 μg of total protein was added to Laemmli sample buffer containing 4% 2-mercaptoethanol and heated to 90 °C for 5 min. The proteins were resolved via SDS−polyacrylamide gel electrophoresis (4−12%) and blotted onto nitrocellulose using an iBlot system (Invitrogen). The PPARγ and Caveolin-1 antibodies were purchased from Santa Cruz Biotechnology Inc. and Cell Signalling Technology, respectively. Uncropped images of western blots are shown in Supplementary Fig. 7.

### Quantification of hepatic triglycerides

Livers were homogenized in triglyceride assay buffer and triglycerides were measured using the Triglyceride quantification Assay Kit (Abcam) according to the manufacturer's instructions.

### Gene expression analysis

Total RNA was isolated from epididymal white adipose tissue using TRIzol reagent (Invitrogen). The RNA was reverse-transcribed using Invitrogen reverse transcription kit. Quantitative PCR (qPCR) reactions were performed with SYBR green fluorescent dye using an ABI9300 PCR machine. Relative mRNA expression was determined by the ΔΔ-*C*_t_ method using glyceraldehyde 3-phosphate dehydrogenase levels. qPCR primers are listed in Supplementary Table 3.

### Histology and immunohistochemistry

Tissues were fixed in 4% paraformaldehyde solution in phosphate buffer for 12 h, placed in 20% sucrose overnight, embedded in Tissue-Tek (SAKURA) and stored at −80 °C until use. For immunofluorescent staining, tissue sections were incubated with anti-insulin antibody (Abcam), followed by a secondary fluorophore-conjugated antibodies (Invitrogen), or with FITC conjugated anti-mouse CD11c (BD Bioscience), or with Cy5 conjugated anti-mouse F4/80 (eBioscience) and counterstained with 4′,6-diamidino-2-phenylindole (DAPI) (Sigma). Islet morphology and eWAT macrophage infiltration were visualized using an Olympus Fluoview F1000 confocal laser-scanning microscope. To investigate the presence of lipids in the liver, sections (8 μM) were stained with Oil Red O (LIFELINE) in accordance with standard procedures. The sections of epididymal white adipose tissue (40 μM) were stained using hematoxylin and eosin. Morphometric analysis of white adipose tissue from 300 cells from 4 different animals per group were performed with NIH ImageJ software (http://rsb.info.nih.gov/ij/).

### Statistical analysis and general methods

To enable statistical significance, five to ten age-matched and weight-matched mice cohort were randomly assigned to treatment or control groups. Statistical analysis (two-tailed student's *t*-test) was applied and *P* values lower than 0.05 were considered significant.

## Additional information

**How to cite this article:** Zhang, H. *et al.* Autocrine selection of a GLP-1R G-protein-biased agonist with potent antidiabetic effects. *Nat. Commun.* 6:8918 doi: 10.1038/ncomms9918 (2015).

## Supplementary Material

Supplementary Information Supplementary Figures 1-7 and Supplementary Table 1-3.

## Figures and Tables

**Table 1:** *In vitro* activity of GLP-1R peptides.

**Peptide**	**Gαs**				**Gαq**		**β-Arrestin 1**		**β-Arrestin 2**	
	**Human GLP-1R**				**Mouse GLP-1R**		**Human GLP-1R**		**Human GLP-1R**		**Human GLP-1R**	
	**EC**_**50**_ **(nM)**	***E***_**max**_ **(%)**	**EC**_**50**_ **(nM)**	***E***_**max**_ **(%)**	**EC**_**50**_ **(nM)**	***E***_**max**_ **(%)**	**EC**_**50**_ **(nM)**	***E***_**max**_ **(%)**	**EC**_**50**_ **(nM)**	**E**_**max**_ **(%)**	
**Ex4**	0.091±0.016	100±1	0.322±0.096	100±1	55.1±26	100±3	29.6±5	100±2	5.6±2	100±7
**GLP-1**	0.912±0.141	73±4	0.623±0.176	89±5	308.5±91	60±8	280.8±86	71±9	60.1±12	77±7
**P1**	2028±234	14±5	542±234	18±7	ND	ND	ND	ND	ND	ND
**P2**	385.6±167	51±16	364.3±138	53±10	ND	ND	ND	ND	ND	ND
**P3**	NC	NC	NC	NC	ND	ND	ND	ND	ND	ND
**P5**	0.852±0.136	102±1	0.967±0.325	100±2	84.9±23	113±11	447.6±134	29±4	529.7±145	32±2
**P10**	3.2±2.7	11±4	NC	5±3	ND	ND	ND	ND	ND	ND

Characterization of the relative efficacy of the peptide to signal through Gαs-protein in human and mouse GLP-1R expressing cell lines, and through Gαq-protein, β-arrestin 1 and -2 in human GLP-1R expressing cell lines. EC_50_ values represent the concentration (nM) of peptide required to stimulate half-maximum GLP-1R activation. *E*_max_ (%) values represent the maximum activation obtained with each peptide relative to Ex4 maximum activation. The data are means±s.d. of a typical experiment that was performed three times. Sequences can be found in Fig. 1e. NC, do not converge; ND, not determined.
