# Supplementary material for: Autocrine selection of a GLP-1R G-protein biased agonist with potent antidiabetic effects
Source: Nat Commun. 2015 Dec 1;6:8918. doi: 10.1038/ncomms9918 (PMC4686834; doi:10.1038/ncomms9918)
Supplement: Supplementary Information — Supplementary Figures 1-7 and Supplementary Table 1-3. [file ncomms9918-s1.pdf]

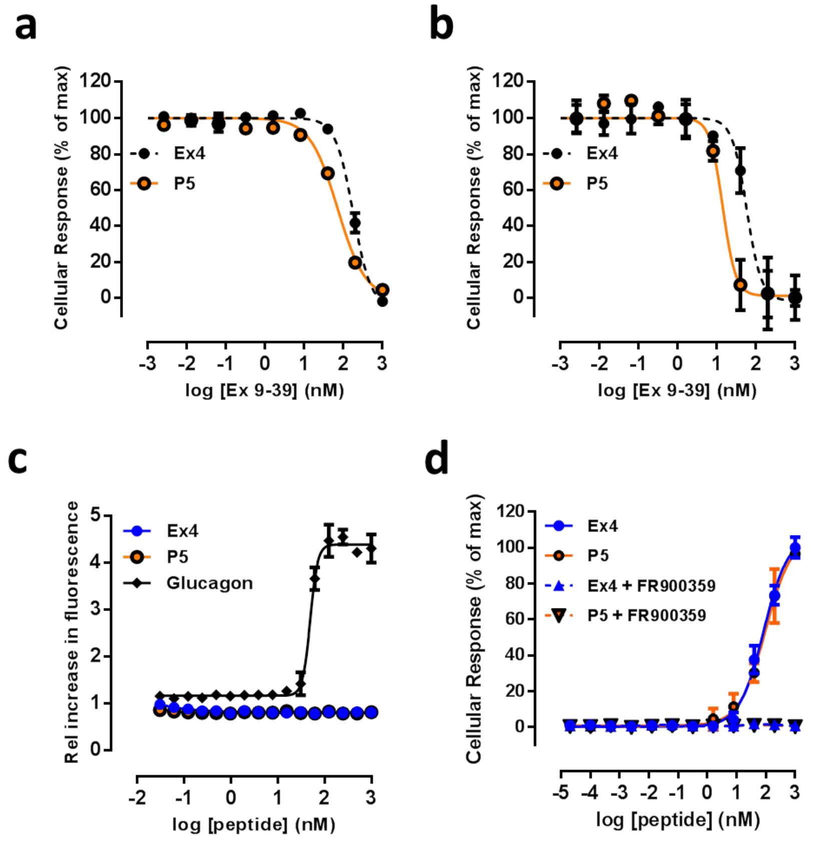

Supplementary Figure 1 *In vitro* pharmacological characterization of P5 selectivity. (**a, b**) Concentration response curves for Ex 9-39 induced decrease in cAMP production in presence of an EC90 of P5 in CHO cells expressing the human GLP-1R (**a**) or in HEK293 expressing the mouse GLP-1R (**b**). (**c**) Concentration response curves for glucagon, P5, and Ex4-induced increase in cAMP production in CHO cells expressing the human glucagon receptor. (**d**) Concentration response curves for P5, and Ex4-induced calcium mobilization in CHO cells expressing the human GLP-1R in the presence or the absence of 1 µM of the specific Gq inhibitor FR900359. The data are mean ± s.e.m. of a typical experiment that was performed independently three times .

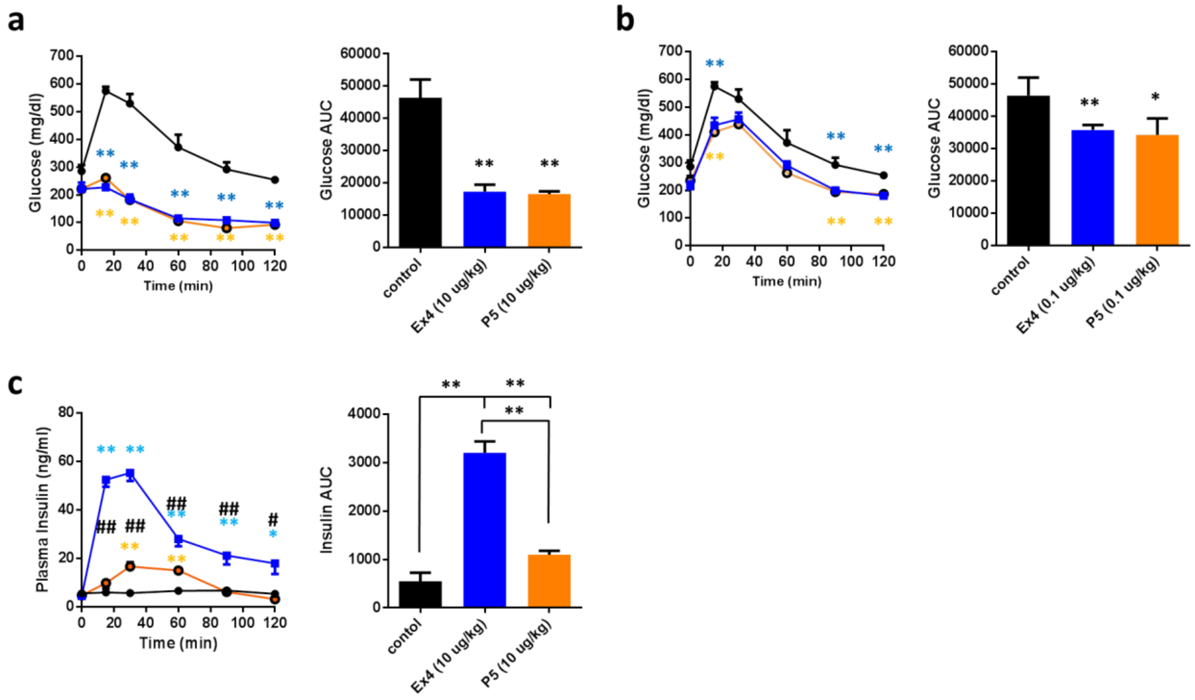

Supplementary Figure 2 Effect of Acute P5 injection on glycaemia and insulin levels in *ob/ob* mice. (**a-c**) Effect on intraperitoneal glucose tolerance and plasma insulin levels following a single intraperitoneal co-injection with glucose challenge (n=5) of saline (black), Ex4 (blue) and P5 (orange). Glucose tolerance in *ob/ob* mice treated with 10 µg/kg (**a**), 0.1 µg/kg (**b**) (n=5)**.** (**c**) Plasma insulin level in *ob/ob* mice were analyzed after a single intraperitoneal co-injection of saline (black), 10 µg/kg of Ex4 (blue) or 10 µg/kg of P5 (orange) with glucose challenge (n=5). Data are mean ± s.e.m. Statistic by two-tailed *t*-test: $^{*}P < 0.05$; $^{**}P < 0.01$, comparing saline to peptide injection; $^{\#}P < 0.05$; $^{\#\#}P < 0.01$, comparing Ex4 to P5 injection. AUC, area under the curve.

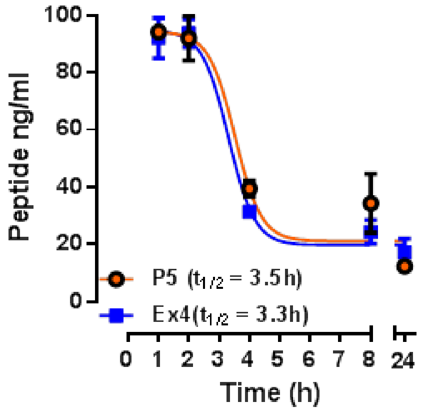

Supplementary Figure 3 Pharmacokinetic profile and half-life of P5 and Ex4 in mice. Peptides were administrated as a single subcutaneous dose at 100 μg/kg. Data are the concentrations of the peptides determined from plasma samples (n=3) by enzyme-linked immunoabsorbent assay. Data are shown as mean ± standard deviation.

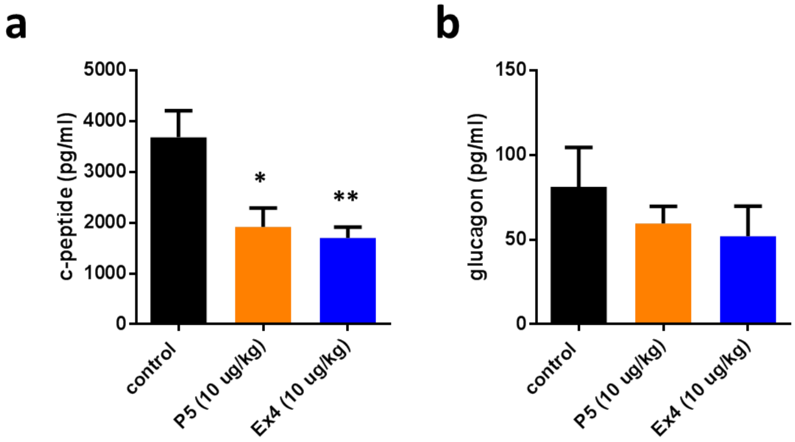

Supplementary Figure 4 Effect of chronic administration of P5, a G-protein biased agonist on metabolic hormones in DIO mice. Four weeks of treatment of DIO male mice with daily dose of Ex4 or P5 biased agonist. Effects on c-peptide plasma level (**a**) and glucagon plasma level (**b**) after daily subcutaneous injections of saline (black), Ex4 (blue) or P5 (orange) at 10 µg/kg (n=8). Data are mean ± s.e.m. Statistic by two-tailed *t*-test: *$P < 0.05$; **$P < 0.01$, comparing saline to peptide injection.

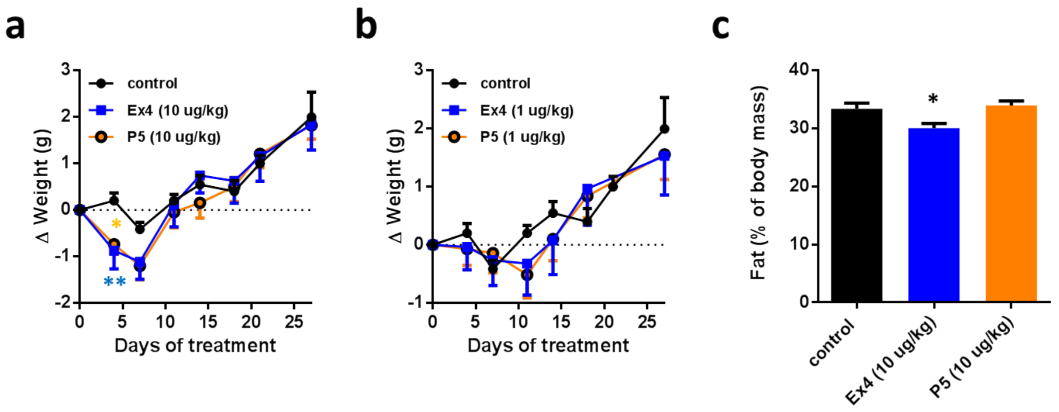

Supplementary Figure 5 Chronic administration of P5, a G-protein biased agonist improves glycemic status in diabetic mice independently of body weight and fat mass. (**a**) Effect on body weight (n=8) after daily subcutaneous injections of saline (black), Ex4 (blue) and P5 (orange) at 10 µg/kg. (**b**) Effect on body weight (n=8) after daily subcutaneous injections of saline (black), Ex4 (blue) and P5 (orange) at 1 µg/kg. (**c**) MRI analyses of fat (n=8) following daily subcutaneous injections of saline (black), Ex4 (blue) and P5 (orange) at 10 µg/kg. Data are mean ± s.e.m. Statistic by two-tailed *t*-test: *$P < 0.05$; **$P < 0.01$, comparing saline to peptide injection.

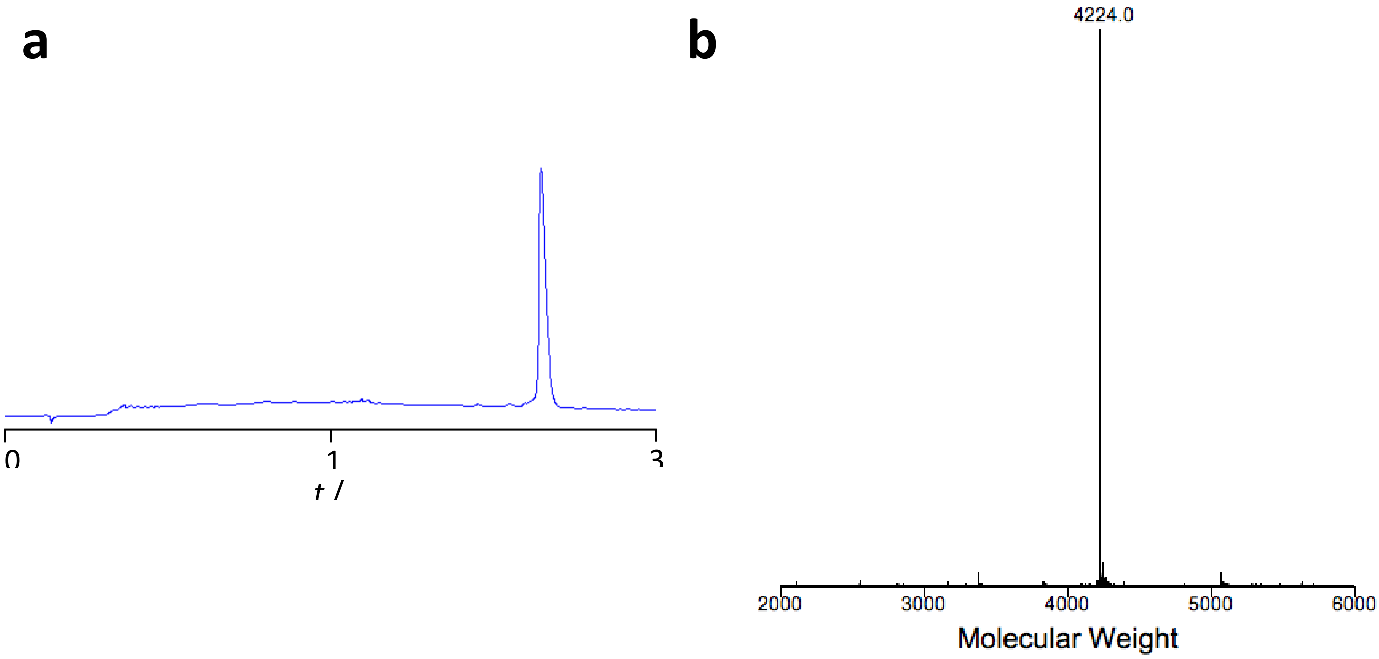

Supplementary Figure 6 (a) Analytical reversed-phase (RP) HPLC was carried out on an Agilent 1100 Series HPLC on a Phenomenex Jupiter Proteo column (4 µm, 90 Å, 150 × 4.6 mm) at a flow rate of 1 mL/min. Using solvents Buffer A (100% H_2O with 0.05% TFA) and Buffer B (90%:10% ACN: H_2O with 0.05% TFA), a 30 minute gradient of 0% Buffer B to 60% Buffer B was ran. An analytical injection containing 50 µg of purified peptide was monitored at 214 nm to determine a final purity of 97.7% by peak area. (**b**) P5 was characterized using electrospray ionization MS on a LC/MS API 2000 Plus triple quadrupole mass spectrometer (Sciex). Peptide masses were calculated from the experimental mass to charge (m/z) ratios from all of the observed protonation states of a peptide by using the onboard analyst software package (Sciex), yielding an observed mass of 4224.0 Da (calculated mass = 4224.6 Da).

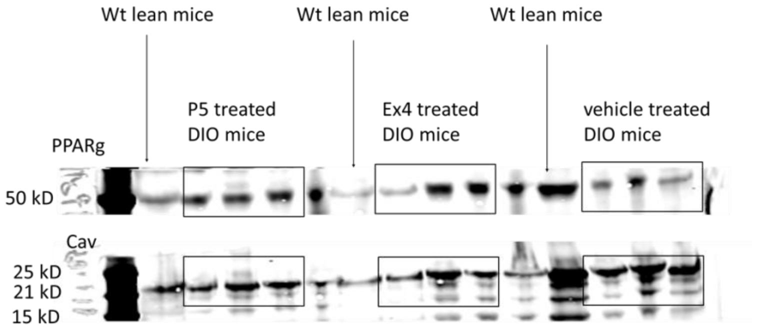

Supplementary Figure 7 Uncropped Licor images of western blots.

	Gαs vs β-arrestin1	Gαs vs β-arrestin2	Gαq vs β-arrestin1	Gαq vs β-arrestin2
**GLP1**	-0.08 ± 0.11	-0.04 ± 0.11	0.16 ± 0.13	0.2 ± 0.08
**P5**	0.9 ± 0.20	1.4 ± 0.27	1.7 ± 0.20	2.4 ± 0.17

Supplementary Table 1 Biased factors for GLP1 and P5. The data are mean ± s.d. of a typical experiment that was performed three times.

	Triglycerides	HDL	LDL
**Treatment**	**mg/dL**	**mg/dL**	**mg/dL**
**control**	115 ± 20	177 ± 27	29 ± 7
**P5 (10ug/kg)**	125 ± 35	189 ± 15	21 ± 3 *
**Ex4 (10ug/kg)**	120 ± 34	186 ± 23	20 ± 4*
**P5 (1ug/kg)**	130 ± 36	173 ± 34	26 ± 10
**Ex4 (1ug/kg)**	113 ± 18	199 ± 31	26 ± 11

Supplementary Table 2 Metabolic parameters of DIO mice chronically treated with P5. Male DIO mice injected subcutaneously daily with vehicle (control), P5, or Ex4 for 4 weeks (n=8). Data are mean ± s.e.m. Statistic by two-tailed *t*-test: *P < 0.05, comparing saline to peptide injection.

Gene	Forward primer	Reverse primer
PPARγ	GCATGGTGCCTTCGCTGA	TGGCATCTCTGTGTCAACCATG
Glut4	GTGACTGGAACACTGGTCCTA	CCAGCCACGTTGCATTGTAG
CD36	AAGCTATTGCGACATGATT	GATCCGACACAGCGTAGAT
TNFα	GACGTGGAAGTGGCAGAAGAG	TGCCACAAGCAGGAATGAGA

Supplementary Table 3 Primer sequences used for qPCR.
